# DKK3 as a potential novel biomarker in patients with autosomal polycystic kidney disease

**DOI:** 10.1093/ckj/sfad262

**Published:** 2023-10-13

**Authors:** Sita Arjune, Martin R Späth, Simon Oehm, Polina Todorova, Stefan J Schunk, Katharina Lettenmeier, Seung-Hun Chon, Malte P Bartram, Philipp Antczak, Franziska Grundmann, Danilo Fliser, Roman-Ulrich Müller

**Affiliations:** Department II of Internal Medicine and Center for Molecular Medicine Cologne, University of Cologne, Faculty of Medicine and University Hospital Cologne, Cologne, Germany; Center for Rare Diseases Cologne, Faculty of Medicine and University Hospital Cologne, University of Cologne, Cologne, Germany; Cologne Excellence Cluster on Cellular Stress Responses in Aging-Associated Diseases (CECAD), Cologne, Germany; Department II of Internal Medicine and Center for Molecular Medicine Cologne, University of Cologne, Faculty of Medicine and University Hospital Cologne, Cologne, Germany; Cologne Excellence Cluster on Cellular Stress Responses in Aging-Associated Diseases (CECAD), Cologne, Germany; Department II of Internal Medicine and Center for Molecular Medicine Cologne, University of Cologne, Faculty of Medicine and University Hospital Cologne, Cologne, Germany; Department II of Internal Medicine and Center for Molecular Medicine Cologne, University of Cologne, Faculty of Medicine and University Hospital Cologne, Cologne, Germany; Department of Internal Medicine IV, Nephrology and Hypertension, Saarland University Medical Center, Homburg/Saar, Germany; Department II of Internal Medicine and Center for Molecular Medicine Cologne, University of Cologne, Faculty of Medicine and University Hospital Cologne, Cologne, Germany; Department of General, Visceral, Cancer and Transplant Surgery, University Hospital of Cologne, Cologne, Germany; Department of Gastroenterology and Hepatology, University Hospital of Cologne, Cologne, Germany; Department II of Internal Medicine and Center for Molecular Medicine Cologne, University of Cologne, Faculty of Medicine and University Hospital Cologne, Cologne, Germany; Department II of Internal Medicine and Center for Molecular Medicine Cologne, University of Cologne, Faculty of Medicine and University Hospital Cologne, Cologne, Germany; Cologne Excellence Cluster on Cellular Stress Responses in Aging-Associated Diseases (CECAD), Cologne, Germany; Department II of Internal Medicine and Center for Molecular Medicine Cologne, University of Cologne, Faculty of Medicine and University Hospital Cologne, Cologne, Germany; Department of Internal Medicine IV, Nephrology and Hypertension, Saarland University Medical Center, Homburg/Saar, Germany; Department II of Internal Medicine and Center for Molecular Medicine Cologne, University of Cologne, Faculty of Medicine and University Hospital Cologne, Cologne, Germany; Center for Rare Diseases Cologne, Faculty of Medicine and University Hospital Cologne, University of Cologne, Cologne, Germany; Cologne Excellence Cluster on Cellular Stress Responses in Aging-Associated Diseases (CECAD), Cologne, Germany

**Keywords:** ADPKD, biomarker, DKK3, prognosis, WNT signalling pathway

## Abstract

**Backgound:**

Autosomal dominant polycystic kidney disease (ADPKD) is the most common inherited kidney disease, and leads to a steady loss of kidney function in adulthood. The variable course of the disease makes it necessary to identify the patients with rapid disease progression who will benefit the most from targeted therapies and interventions. Currently, magnetic resonance imaging–based volumetry of the kidney is the most commonly used tool for this purpose. Biomarkers that can be easily and quantitatively determined, which allow a prediction of the loss of kidney function, have not yet been established in clinical practice. The glycoprotein Dickkopf 3 (DKK3) which is secreted in the renal tubular epithelium upon stress and contributes to tubulointerstitial fibrosis via the *Wnt* signaling pathway, was recently described as a biomarker for estimating risk of kidney function loss, but has not been investigated for ADPKD. This study aimed to obtain a first insight into whether DKK3 may indeed improve outcome prediction in ADPKD in the future.

**Methods:**

In 184 ADPKD patients from the AD(H)PKD registry and 47 healthy controls, the urinary DKK3 (uDKK3) levels were determined using ELISA. Multiple linear regression was used to examine the potential of these values in outcome prediction.

**Results:**

ADPKD patients showed significantly higher uDKK3 values compared with the controls (mean 1970 ± 5287 vs 112 ± 134.7 pg/mg creatinine). Furthermore, there was a steady increase in uDKK3 with an increase in the Mayo class (A/B 1262 ± 2315 vs class D/E 3104 ± 7627 pg/mg creatinine), the best-established biomarker of progression in ADPKD. uDKK3 also correlated with estimated glomerular filtration rate (eGFR). Patients with *PKD1* mutations show higher uDKK3 levels compared with *PKD2* patients (*PKD1*: 2304 ± 5119; *PKD2*: 506.6 ± 526.8 pg/mg creatinine). Univariate linear regression showed uDKK3 as a significant predictor of future eGFR slope estimation. In multiple linear regression this effect was not significant in models also containing height-adjusted total kidney volume and/or eGFR. However, adding both copeptin levels and the interaction term between copeptin and uDKK3 to the model resulted in a significant predictive value of all these three variables and the highest R^2^ of all models examined (∼0.5).

**Conclusion:**

uDKK3 shows a clear correlation with the Mayo classification in patients with ADPKD. uDKK3 levels correlated with kidney function, which could indicate that uDKK3 also predicts a disproportionate loss of renal function in this collective. Interestingly, we found an interaction between copeptin and uDKK3 in our prediction models and the best model containing both variables and their interaction term resulted in a fairly good explanation of variance in eGFR slope compared with previous models. Considering the limited number of patients in these analyses, future studies will be required to confirm the results. Nonetheless, uDKK3 appears to be an attractive candidate to improve outcome prediction of ADPKD in the future.

KEY LEARNING POINTS **What was known:**

- The prognosis of autosomal dominant polycystic kidney disease (ADPKD) progression is difficult and is currently based on markers requiring sophisticated diagnostics such as total kidney volume and genotype.
- The discovery of novel biomarkers and risk factors is critical to facilitate accurate prognostic statements and improve patient selection for targeted, costly and side effect–prone treatments.

**This study adds:**

- The study aims to determine the potential role of urinary Dickkopf 3 (uDKK3) as a novel, disease-predicting biomarker in ADPKD.
- The study found a strong correlation of uDKK3 with both age and estimated glomerular filtration rate in ADPKD patients.
- Increasing median uDKK3 was observed in ADPKD patients with increasing chronic kidney disease stages.
- Adding uDKK3 to current models of disease progression in ADPKD may improve their accuracy, especially when combined with copeptin.

**Potential impact:**

- While this study will need confirmation in larger cohorts it already identifies uDKK3 as a promising biomarker.
- The results can now be the basis to setting up larger prospective studies.
- The study also indicates that the combination of novel biomarkers is a promising way to improve outcome prediction in ADPKD.

## INTRODUCTION

Autosomal dominant polycystic kidney disease (ADPKD) is the most common inherited kidney disease and the fourth leading cause of kidney failure worldwide [1]. Cyst formation and progressive decline in kidney function are the primary consequences of mutations in PKD genes (primarily *PKD1* or *PKD2*) [2]. By the age of 70 years, more than 70% of patients develop kidney failure [3]. However, even within families sharing the same mutations, the interindividual disease course is highly variable, making future outcome prediction difficult. Two randomized clinical trials established the efficacy of tolvaptan, a V_2_R antagonist, resulting in the approval of the first disease-modifying drug [4]. Since only patients with rapidly progressive disease benefit from tolvaptan treatment, and because tolvaptan has significant side effects (most notably polyuria and polydipsia), patient selection for reasonable treatment recommendations has become critical. Predicting disease progression in ADPKD, however, remains a difficult task. Predictive markers of progression that are currently available, such as genetics, past-time estimated glomerular filtration rate (eGFR) loss and age-adapted total kidney volume (TKV) using the Mayo classification, are useful in routine clinical care but are limited by various factors, such as the requirement for magnetic resonance imaging (MRI) scans or the absence of past-time creatinine measurements [5–7]. Therefore, the identification of novel and easily accessible biomarkers will help to identify the subgroup of patients that benefit the most regarding targeted therapies and will improve patient counseling. Additionally, new biomarkers could allow for the prediction of long-term treatment responses. The glycoprotein Dickkopf 3 (DKK3) is secreted in the renal tubular epithelium under stress and contributes to tubulointerstitial fibrosis via the *Wnt* signaling pathway. DKK3 was recently described as a biomarker for estimating a loss of renal function of various origins and has now been investigated for the first time in patients with ADPKD [8]. The current study sought to determine whether urinary DKK3 (uDKK3) is a potential biomarker in an ADPKD real-world scenario and whether it can improve the predictive accuracy of currently used clinical criteria.

## MATERIALS AND METHODS

### Patient population

In this study 184 adult (≥18 years) ADPKD patients who were enrolled in the AD(H)PKD registry and 47 healthy controls (German Clinical Trials Register: DRKS00014637) were included in the analysis. The AD(H)PKD registry comprises tolvaptan-naïve patients and patients starting therapy after baseline visit as well as patients already on targeted therapy. Follow-up visits were conducted for all patients and included yearly measurements of serum creatinine and other laboratory parameters (e.g. serum osmolality, urine osmolality). To exclude an infection that interferes with the measurement of uDKK3, individuals with elevated C-reactive protein (CRP) levels or signs of urinary tract infection were excluded from further analyses. Written informed consent was obtained from all patients and approval was retrieved from the institutional review board of the University of Cologne. The cohort study was conducted following the Declaration of Helsinki and the good clinical practice guidelines by the International Conference on Harmonization registered on www.ClinicalTrials.gov (NCT02497521).

### Data collection, uDKK3 measurements and descriptions

Clinical and laboratory parameters of all patients were available from the AD(H)PKD registry. TKV at baseline was assessed using standardized kidney MRI and manual segmentation, while TKV follow-up measurements were not regularly performed. At all visits, patients were advised to present in a fasted state. uDKK3 measurement was performed at a single visit (either baseline and follow-up visits) using an ELISA-based assay described previously [9]. In brief, prior to measurement, urine samples were clarified by centrifugation to remove debris that may interfere with the assay. All urine samples were immediately frozen at –80°C after collection, and additional analyses showed no relevant degradation of DKK3 during freezing. To account for dilution of urine, uDKK3 levels were normalized to urinary creatinine concentrations. To minimize potential bias from different sample processing, DKK3 measurements in all samples from all cohorts presented in the manuscript were performed at one site (Saarland University Medical Center) in a blinded manner. Intra-assay test variabilities for repeated urine sample measurements are 3.1% in the lower detection range (approximately 500 pg DKK3 per 1 mL urine) and 3.5% in the higher detection range (approximately 1500 pg DKK3 per 1 mL urine) [9]. Inter-assay test variability is 4.7% in the lower detection range and 5.1% in the higher detection range [9].

Data collection was completed by obtaining eGFR values from outpatient nephrologists or general practitioners with written informed consent available for all patients. eGFR was calculated using the Chronic Kidney Disease Epidemiology Collaboration equation 2009 [10].

### Linear modeling of disease outcomes

Baseline eGFR values were developed using historic creatinine measurements up to 7071 days prior and 1642 days after uDKK3 measurements (Supplementary data, Fig. S1). For each patient with at least five creatinine measurements, an eGFR slope was calculated. Where applicable and when sufficient data were available, a tolvaptan-specific slope was additionally generated. The models were developed using a robust linear modeling approach as implemented in the MASS library. These models followed the simple equation shown in Eqn (1) where Δdate was the difference in days between the baseline uDKK3 measurement and the current creatinine measurement.


(1) \begin{equation*}{\mathrm{eGFR}} = {\mathrm{\ \Delta date}}\end{equation*}

Slopes were then extracted to be the parameter associated with Δdate (differences in days) and multiplied by 365 to retrieve an annual eGFR slope. eGFRs predicted for the specific dates at which uDKK3 has been measured were then calculated and used in subsequent modeling approaches.

The resulting slope was then used in a linear regression to develop the Models I–X. The selection of parameters was guided by clinical considerations that underlie the disease. Each model was carefully chosen based on prior clinical understanding and uDKK3 added to test whether it significantly contributes to a model of disease progression. Given that uDKK3 and copeptin, on their own, explained some of the variation observed in the eGFR slopes and the addition of both of these parameters significantly improved the association, an interaction between these two was added in the final models (IX and X).

### Statistics

Baseline patients’ characteristics are reported as mean ± standard deviation (SD) for normal distributions and median [interquartile range (IQR)] for skewed distributions. Data were tested for normality using the Shapiro–Wilk test. The *P*-values for statistical difference were computed using paired Student's *t*-test for normally distributed data and Wilcoxon signed-rank test for uDKK3 values. Analysis of variance (ANOVA) was employed to assess the statistical significance of differences in means among three or more groups. Multiple testing was corrected for using the Bonferroni method to control the family-wise error rate at the specified significance level. uDKK3 values were analyzed for both their distribution in different patient groups (e.g. age, Mayo class) as well as their response to tolvaptan treatment. A *P-*value <.05 was considered to be statistically significant. All analyses were performed using R (Version 4.0.3) with the libraries MASS for robust linear models.

## RESULTS

### Demographics and clinical characteristics of the study participants

**Figure 1:**
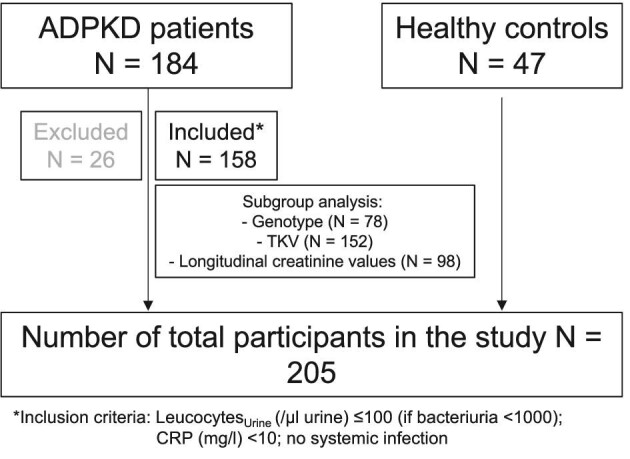
Study flow chart illustrating patient flow, inclusion criteria, as well as subgroup analyses.

This study initially included 184 ADPKD patients enrolled in the AD(H)PKD registry, of which 158 adult ADPKD patients (84 men and 74 women) were selected after the exclusion of 26 patients (Fig. 1). The mean age of the ADPKD patients was 44.4 ± 11.0 years (range 18–73). In addition, 47 healthy individuals (22 men and 25 women) were included as a control in the analysis. The mean age of the control group was 46.0 ± 16.2 years (range 20–74). The results are summarized in Table 1.

**Table 1:** Cohort characteristics.

Characteristics	ADPKD	Controls
Subjects	158	47
Age (years), mean ± SD	44.4 ± 11.0	46.0 ± 16.2
eGFR (mL/min/1.73 m^2^), mean ± SD	62.0 ± 29.0	
Total kidney volume (mL), mean ± SD	2116 ± 1716	
Without baseline MRI, *n*	6	
Mayo classification, *n*	152	
1A	1	
1B	30	
1C	53	
1D	45	
1E	23	
CKD stage, *n*	158	
1	31	
2	42	
3	66	
4	17	
5	2	
Positive family history for ADPKD, *n* (%)	136 (86.07)	
Hypertension, *n*	142	
<35 years of age	74	
≥35 years of age	68	
Urological complications, *n*	100	
<35 years of age	52	
≥35 years of age	48	
Tolvaptan intake, *n* (%)	21 (13.39)	
Genotype, *n*	78	
*PKD1*, truncating	14	
*PKD1*, non-truncating	46	
*PKD2*	18	
PROPKD score, *n*	78	
Low (0–3)	31	
Medium (4–6)	27	
High (7–9)	20	

**Figure 2:**
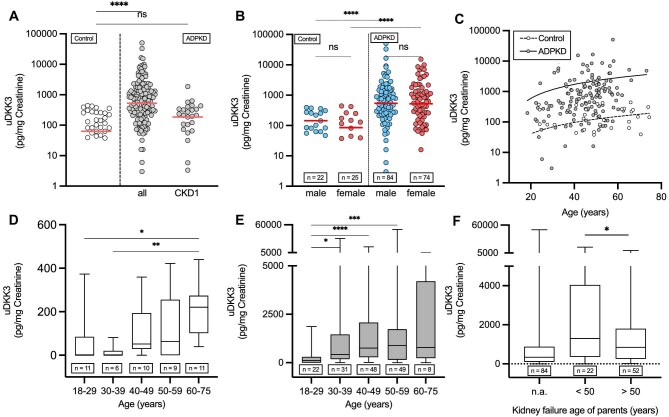
uDKK3 levels in ADPKD patients (*n* = 158, uDKK3 median 528 pg/mg, IQR 1523–170.5 pg/mg; CKD 1 *n* = 31, uDKK3 median = 185.1 pg/mg, IQR 335.4–5.860 pg/mg) and healthy controls (uDKK3 median 63.00 pg/mg, IQR 221–0 pg/mg) (**A**) and in association to gender (**B**) [ADPKD: male (*n* = 84), median 540.00 pg/mg, IQR 1582–215.3 pg/mg; female (*n* = 74), media 521.50 pg/mg, IQR 1484–110.3 pg/mg; Control: male (*n* = 22): 84.33 pg/mg, IQR 261.1–35.45 pg/mg; female 0 pg/mg, IQR 139.2–0 pg/mg] and age (C–E) [(**C**) Spearman correlation, ADPKD (continuous line): r = 0.2544, *P* = .0013, Control (dashed line): r = 0.4285, *P* = .0027, (**D**) median: 18–29: 0 pg/mg, IQR 85.00–0 pg/mg; 30–39 years: 0 pg/mg, IQR 20.25–0 pg/mg; 40–49 years: 51.50 pg/mg, IQR 194.0–28.5 pg/mg; 50–59 years: 63.00 pg/mg, IQR 255.0–0 pg/mg; 60–75 years: 221.0 pg/mg, IQR 274.0–102.0 pg/mg, (**E**) median: 18–29 years: 114.5 pg/mg, IQR 302.0–2.25 pg/mg; 30–39 years: 430.0 pg/mg, IQR 1459–185.0 pg/mg; 40–49 years: 754.5 pg/mg, IQR 2077–286.8 pg/mg; 50–59 years: 889.9 pg/mg, IQR 1730–151.0 pg/mg; 60–75 years: 792.5 pg/mg, IQR 4204–239.5 pg/mg] and the onset of dialysis with (**F**) (median: n.a. 340.5 pg/mg, IQR 877.8–105.8 pg/mg; <50: 1298 pg/mg, IQR 4042–354.8 pg/mg; >50: 847.5 pg/mg, IQR 1801–261.8 pg/mg). Error bars indicate SD. ANOVA was performed for (A), (B), (D), (E) and (F). ****P < .0001, ***P < .001, **P < .01, *P < .05, ns indicates not significant. Direct comparison of different age groups for ADPKD patients and controls can be found in Supplementary data, Fig. S2.

To obtain insight into the distribution of uDKK3 levels in our cohort, we examined clinical factors known to be associated with a modulation of uDKK3. ADPKD patients showed significantly higher uDKK3 levels (528.5 pg/mg creatinine, median) than healthy controls (63 pg/mg creatinine, median) (Fig. 2A), while no gender-specific effect was found (Fig. 2B). uDKK3 positively correlated with the age of ADPKD patients (Fig. 2C). While uDKK3 levels increased significantly with age (*P* = .0013) in both ADPKD patients and healthy controls, the increase was less pronounced in healthy controls (*P* = .0027) (Fig. 2C). The age-specific difference in uDKK3 levels showed a linear trend towards higher values in older ADPKD patients and healthy controls (Fig. 2D and E). The results of these analyses confirm the significant difference in uDKK3 levels between age groups and support the notion that healthy controls exhibit a less pronounced increase than ADPKD patients. For uDKK3 levels, we also performed a linear regression analysis including an interaction term between cohort (ADPKD versus control) and age (Supplementary data, Table S1). The analysis reveals a significant interaction effect between cohort and age on uDKK3 concentrations (age: control, *P* = .0407). This demonstrates that the correlation between age and uDKK3 levels differs between the ADPKD and control groups, confirming our initial findings. Furthermore, uDKK3 levels were significantly higher in ADPKD patients who had an affected parent with an onset of kidney failure before the age of 50 years (Fig. 2F).

**Figure 3:**
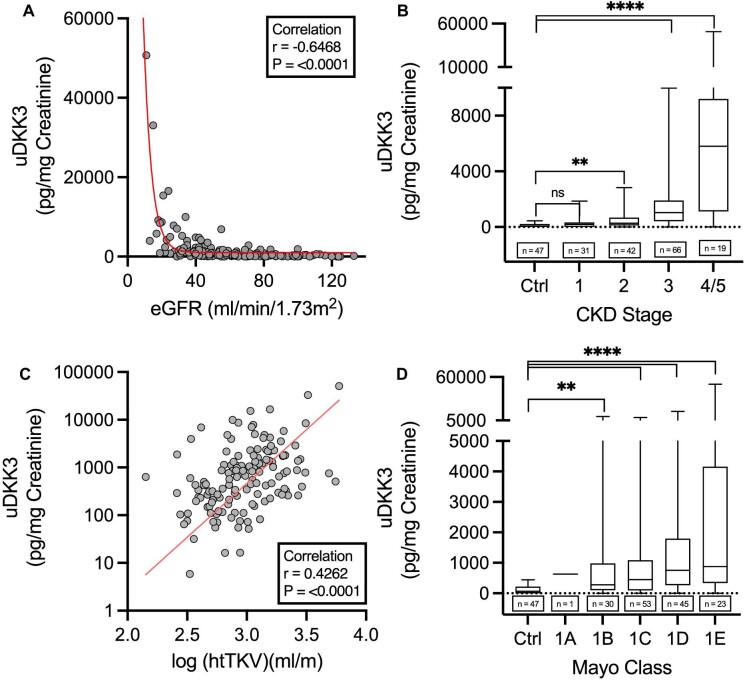
Correlation of uDKK3 levels in ADPKD patients with eGFR (**A**) and htTKV (**C**) and according to CKD stages (**B**) (median: control: 63.0 pg/mg, IQR 221.0–0 pg/mg; CKD 1: 185.0 pg/mg, IQR 335.0–6.0 pg/mg; CKD 2: 273.5 pg/mg, IQR 678.3–111.8 pg/mg; CKD 3: 1039 pg/mg, IQR 1898–413.0 pg/mg; CKD 4/5: 5792 pg/mg, IQR 9197–1121 pg/mg) and Mayo classes (**D**) (median: control: 63 pg/mg, IQR 221.0–0 pg/mg; 1A: 631.0 pg/mg; 1B: 280.0 pg/mg, IQR 982.3–104.8 pg/mg; 1C: 450.0 pg/mg, IQR 1084–98.50 pg/mg; 1D: 757.0 pg/mg, IQR 1798–265.5 pg/mg; 1E: 880.0 pg/mg, IQR 4155–339.0 pg/mg). Spearman correlation was conducted after the D'Agostino and Pearson test revealed non-parametric data distribution. Non-linear curve fitting was achieved by using a one-phase exponential decay equation for eGFR and an exponential growth equation for htTKV. ****P < .0001, ***P < .001, **P < .01, *P < .05, ns indicates not significant.

uDKK3 showed a strong negative correlation with eGFR (Fig. 3A) and consequently, a positive linear trend of higher uDKK3 levels with higher chronic kidney disease (CKD) stages was found (Fig. 3B). A similar trend was seen for height-adjusted total kidney volume (htTKV) and Mayo classes (Fig. 3C and D), a classification used to assess the risk of progression based on the patient's htTKV and age [6].

**Figure 4:**
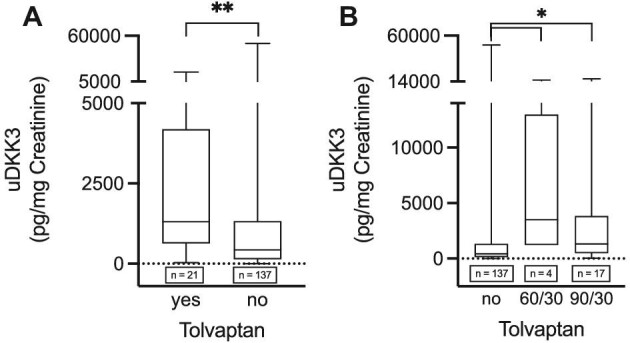
Treatment of tolvaptan leads to an increase in uDKK3 levels. uDKK3 levels in patients with and without tolvaptan (**A**) (median: yes: 1308 pg/mg, IQR 4191–631.5 pg/mg; no: 430 pg/mg, IQR 1326–135.0 pg/mg) and different dosages of tolvaptan (**B**) (median: no: 430 pg/mg, IQR 1326–135.0 pg/mg; 60/30: 3504 pg/mg, IQR 12 980–1212 pg/mg; 90/30: 1308 pg/mg, IQR 3847–480.0 pg/mg). Mann–Whitney test (A) or ANOVA (B) was performed as indicated. Error bars indicate SD. ****P < .0001, ***P < .001, **P < .01, *P < .05, ns indicates not significant.

uDKK3 levels were significantly higher in ADPKD patients taking tolvaptan than in participants not taking tolvaptan at the time of sampling (Fig. 4A, median uDKK3 level 1308 pg/mg creatinine vs 430.0 pg/mg creatinine). This difference was observed both at 60/30 and 90/30 mg of tolvaptan (Fig. 4B).

**Figure 5:**
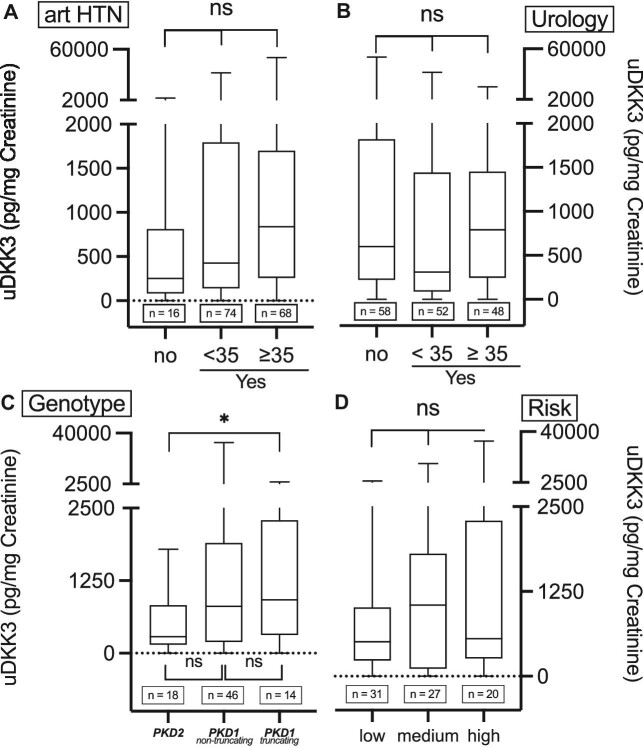
uDKK3 and clinical criteria for rapid disease progression in ADPKD. uDKK3 levels concerning the onset of hypertension (**A**) (median: no: 252.4 pg/mg, IQR 811.2–82.93 pg/mg; <35: 426.8 pg/mg, IQR 1794–139.0 pg/mg; ≥35: 838.1 pg/mg, IQR 1700–258.3 pg/mg) and urological complications (**B**) (median: no: 600.5 pg/mg, IQR 1821–221.8 pg/mg; <35: 310.0 pg/mg, IQR 1443–87.50 pg/mg; ≥35 791.0 pg/mg, IQR 1453–243.8 pg/mg). uDKK3 levels among respective genotypes (*PKD1* and *PKD2*) (**C**) (median: *PKD2*: 286.0 pg/mg, IQR 828.3–145.8 pg/mg; *PKD1* non-truncating: 806.0 pg/mg, IQR 1898–194.3 pg/mg; *PKD1* truncating: 919.5 pg/mg, IQR 311.5–2291 pg/mg). Truncating mutations included nonsense, frameshift, splicing mutations and large rearrangements; non-truncating mutations included missense mutations and in-frame short deletions and insertions. (**D**) Risk of progression to ESRD was determined using the calculated PROPKD score (low = 0–3, median 510.0 pg/mg, IQR 1015–232.0 pg/mg; medium = 4–6, median 1051.0 pg/mg, IQR 1806–112.0 pg/mg; high = 7–9, median 553.0 pg/mg, IQR 2294–261.8 pg/mg). Error bars indicate SD. **** *P* < .0001, ***P < .001, **P < .01, *P < .05, ns indicates not significant.

Furthermore, we assessed whether uDKK3 levels were associated with factors known to be indicative of rapid progression in ADPKD patients. No significant differences were detected in groups with early or late-onset of arterial hypertension (Fig. 5A) and urological complications (including hematuria, cyst infection, flank pain and kidney stones, Fig. 5B). Genetic information was available for 78 ADPKD patients. Truncating *PKD1* variants showed a tendency towards higher uDKK3 levels compared with both non-truncating *PKD1* and *PKD2* variants.

The PROPKD score which further integrates gender, early onset of hypertension and urological complications, and genetic variant type, has been shown to be a predictor of kidney survival [11]. uDKK3 levels were elevated with increasing PROPKD score, although this did not reach statistical significance (Fig. 5D).

We used linear regression models to analyze whether uDKK3 could serve as an independent variable to explain kidney function loss in addition to htTKV and different clinical variables. We used 1631 available creatinine values from 98 patients in these analyses. In summary, a range of 4 values per patient up to 41 values per patient was available (median 15 values per patient, mean 16.47 values per patient). The average duration of follow-up was 1.7 years (median 1.6 years and maximum 4.5 years).

First, we confirmed that uDKK3 is a significant factor by examining a model that uses uDKK3 to explain the eGFR slope (R^2^ = 0.17, Model I, Table 2). Using htTKV to explain the eGFR slope confirmed the significance of htTKV (R^2^ = 0.13, Model II, Table 2). Addition of uDKK3 resulted in a slightly higher R^2^ (R^2^ = 0.16, Model III, Table 2), but only htTKV remained significant as an independent variable. Potentially combining blood parameter–based biomarkers could have added benefit. We further examined the models by adding the biomarker copeptin, which has been shown to be an independent predictor of eGFR slope [12]. Here, copeptin alone served as an independent predictor (Model IV) and addition of copeptin did improve the respective models (Table 3).

**Table 2:** Linear regression models for eGFR slope prediction (*n* = 98).

Model I: eGFR slope ∼ uDKK3				
Adjusted R^2^ 0.17				
Variable	Estimate	SE	*P*-value	*P*-value summary
uDKK3	–1.6758	0.5077	.00187	**
Model II: eGFR slope ∼ htTKV				
Adjusted R^2^ 0.13				
Variable	Estimate	SE	*P*-value	*P*-value summary
htTKV	–1.6024	0.4012	.000127	***
Model III: eGFR slope ∼ htTKV + uDKK3				
Adjusted R^2^ 0.16				
Variable	Estimate	SE	*P*-value	*P*-value summary
htTKV	–1.3757	0.4140	.00127	**
uDKK3	–0.7358	0.3915	.06327	ns

SE, standard error. **** *P* < .0001, *** *P* < .001, ** *P* < .01, * *P* < .05, ns indicates not significant.

**Table 3:** Linear regression models for eGFR slope prediction (*n* = 45).

Model IV: eGFR slope ∼ copeptin				
Adjusted R^2^ 0.28				
Variable	Estimate	SE	*P*-value	*P*-value summary
Copeptin	–1.7647	0.4049	7.28e–05	***
Model V: eGFR slope ∼ uDKK3 + copeptin				
Adjusted R^2^ 0.32				
Variable	Estimate	SE	*P*-value	*P*-value summary
uDKK3	–0.9970	0.5046	.0543	ns
Copeptin	–1.4180	0.4301	.0019	**
Model VI: eGFR slope ∼ htTKV + uDKK3 + copeptin				
Adjusted R^2^ 0.30				
Variable	Estimate	SE	*P*-value	*P*-value summary
htTKV	0.1606	0.8392	.8491	ns
uDKK3	–1.2202	0.5306	.0264	*
Copeptin	–1.2681	0.6501	.0577	ns
Model VII: eGFR slope ∼ htTKV + uDKK3 + eGFR + copeptin				
Adjusted R^2^ 0.33				
Variable	Estimate	SE	*P*-value	*P*-value summary
htTKV	0.13664	0.82 044	.8685	ns
uDKK3	–0.81733	0.56 846	.1579	ns
eGFR	0.03806	0.02 198	.0907	ns
Copeptin	–0.79194	0.69 247	.2592	ns
Model VIII: eGFR slope ∼ uDKK3 + eGFR + age + gender + copeptin				
Adjusted R^2^ 0.38				
Variable	Estimate	SE	*P*-value	*P*-value summary
uDKK3	–0.44838	0.53981	.4109	ns
eGFR	0.04715	0.02475	.0636	ns
Age	0.06227	0.04018	.1287	ns
Gender (female)	–0.92081	0.72042	.2082	ns
Copeptin	–1.11551	0.60203	.0709	ns

SE, standard error. **** *P* < .0001, *** *P* < .001, ** *P* < .01, * *P* < .05, ns indicates not significant.

The addition of copeptin resulted in a significant improvement in the adjusted R^2^ values (Model III → Model VI: R^2^ 0.16 to 0.30). However, upon addition of copeptin to the linear regression models, none of the tested variables remained independent predictors. Intriguingly, when adding the interaction term of both uDKK3 and copeptin (Table 4), the effect of the interaction term on eGFR slope was found to be statistically significant, indicating that the combined effect of these variables differs from what would be predicted by adding their individual effects. In both models, the inclusion of the uDKK3 and copeptin interaction term increased the adjusted R^2^ value (Model VII R^2^ 0.33 → 0.47 and Model VIII R^2^ 0.38 → 0.4991) and both uDKK3 and copeptin remained statistically significant in addition to their interaction term.

**Table 4:** Linear regression models for eGFR slope prediction from interaction terms (*n* = 45).

Model IX: eGFR slope ∼ uDKK3 * copeptin + htTKV + age + gender				
Adjusted R^2^ 0.4707				
Variable	Estimate	SE	*P*-value	*P*-value summary
DKK3	–3.85608	1.29433	.00489	**
Copeptin	–4.99521	1.57949	.00298	**
uDKK3 * copeptin	1.15143	0.46931	.01860	*
htTKV	–0.39732	0.81685	.62933	ns
Age	0.03935	0.03623	.28386	ns
Gender (female)	–1.07462	0.69677	.13088	ns
Model X: eGFR slope ∼ uDKK3 * copeptin + eGFR + age + gender				
Adjusted R^2^ 0.4991				
Variable	Estimate	SE	*P*-value	*P*-value summary
DKK3	–4.13492	1.19 641	.001289	**
Copeptin	–5.41262	1.38 490	.000341	***
uDKK3 * copeptin	1.45 215	0.43 107	.001654	**
eGFR	0.05 870	0.02 243	.012382	*
Age	0.07 198	0.03 610	.052876	ns
Gender (female)	–0.57081	0.65 361	.387572	ns

SE, standard error. **** *P* < .0001, *** *P* < .001, ** *P* < .01, * *P* < .05, ns indicates not significant.

## DISCUSSION

Predicting ADPKD progression is difficult and is currently based on markers requiring MRI such as TKV. The discovery of novel biomarkers and risk factors is critical to facilitate accurate prognostic statements and improve patient selection for targeted, costly and side effect–prone treatments like tolvaptan. Additionally, new biomarkers should enable the estimation of treatment response over time without cost-intensive diagnostics like MRI. The purpose of this study was to determine the potential role of uDKK3 as a novel, disease-predicting biomarker in ADPKD. DKK3 (molecular weight 38 kDa) is a secreted glycoprotein that is synthesized by stressed tubular epithelia and expressed *in vitro* in mesenchymal progenitor and mesenchymal cells [13, 14]. Prior to secretion, DKK3 is substantially glycosylated [15], thereby increasing its molecular weight to 60–70 kDa.

As a multifunctional protein, DKK3 participates in a variety of cellular processes via the *Wnt*/β-catenin pathway including cell differentiation, proliferation and apoptosis [16]. The pathway plays a role in a variety of diseases, including cancer [17, 18] and chronic heart failure [19]; furthermore, it is a critical signaling axis that contributes to kidney disease [20].

While alterations of *Wnt* signaling result in multiple kidney cysts in a mouse model [21], another study found a potential role of genetic variations of DKK3 in ADPKD [8]. By examining three single-nucleotide polymorphisms at the *dkk3* gene locus, a significant association with eGFR was found, thereby indicating DKK3 to potentially modify kidney disease severity in ADPKD [8].

However, a potential role of DKK3 as a biomarker in ADPKD remains unknown. It would be intriguing to speculate that DKK3 may indeed reflect the tendency towards increased fibrosis, one of the mechanisms leading to kidney failure in ADPKD that is not well-reflected by currently available biomarkers. Hence, the purpose of this study was to ascertain the potential role of DKK3 as a potential biomarker in the prediction of disease progression in ADPKD.

Similar to Piek *et al.* [22], we were able to demonstrate a strong correlation between DKK3 and both age and eGFR. While our study utilized urine from ADPKD patients, Piek *et al.* examined plasma from a large general population cohort. A correlation between uDKK3 levels and kidney function was also observed in a cohort of 2314 patients with chronic obstructive pulmonary disease [23]. In these patients, uDKK3 predicted significant loss of eGFR (>20%) within an observational period of 18 months, even in individuals with normal baseline kidney function, i.e. eGFR >90 mL/min/1.73 m^2^, and no albuminuria [23]. In the present study, increasing median uDKK3 were observed in ADPKD patients with increasing CKD stages. While in our control cohort, the median uDKK3 value was higher than in a previously published study (median 63 pg/mg creatinine in this study vs 33 pg/mg creatinine [9]), significantly higher uDKK3 levels were detected in ADPKD patients. In ADPKD, male patients tend to show a faster age-dependent eGFR decline, and thus male gender is viewed as a risk factor for disease progression [24–26]. While other biomarkers [12] tend to map this trend among genders, uDKK3 showed no sex-dependent difference. A linear trend towards higher uDKK3 values with increasing age and hence expected loss of renal function was detected in both groups, however the correlation was more pronounced in the ADPKD group.

Further investigation into the effect of tolvaptan on uDKK3 levels revealed significantly elevated uDKK3 levels in the presence of tolvaptan. However, the different distribution of ADPKD patients with and without tolvaptan regarding CKD stage and Mayo classes must be considered (Supplementary data, Fig. S3). Considering the tolvaptan approval criteria, ADPKD patients on tolvaptan therapy had higher CKD stages and Mayo classes on average.

To obtain a first insight into whether high DKK3 levels may indicate rapid progression, we investigated the association of uDKK3 levels with known progression factors. In the specific context of ADPKD, uDKK3 positively correlated with increasing htTKV and Mayo classes, indicating higher values with disease progression. However, neither early onset of arterial hypertension or urological complications nor risk of progression to end-stage renal disease (ESRD) showed significant differences among the groups. Interestingly, the age at which patients’ parents experienced kidney failure significantly distinguished low and high uDKK3 values.

When testing for genetic differences among ADPKD patients, significant differences were found. ADPKD is genetically heterogeneous with two genes being responsible for the majority of mutations: *PKD1* encoding polycystin-1 (around 72%–75% of cases) and *PKD2* encoding polycystin-2 (around 15%–18% of cases) [27, 28]. Although *PKD1* mutations generally resulted in higher uDKK3 levels than *PKD2*, this difference did not reach statistical significance. However, protein-truncating *PKD1* mutations are generally linked to more severe cases of ADPKD, and DKK3 values were significantly higher in patients with this mutation. DKK3 is a member of an evolutionarily conserved gene cluster that is active during developmental processes, further silenced, and re-expressed during disease states [14].

A recent study [9] described the association between uDKK3 and tubulointerstitial fibrosis in a cohort of patients undergoing diagnostic kidney biopsy and in kidney biopsy specimens of Supportive Versus Immunosuppressive Therapy for the Treatment of Progressive IgA Nephropathy (STOP-IgAN) trial participants [29]. Interestingly, uDKK3 concentrations were significantly associated (*P* < .001) with higher-grade tubulointerstitial fibrosis in the biopsy specimens of both groups, indicating a potential role of DKK3 in both glomerular and interstitial diseases [9]. Hence, in ADPKD sustained tubular stress caused by extensive cysts growth may result in tubular excretion of DKK3. However, as ADPKD is a disease that affects multiple systems and DKK3 is expressed in various organs with measurable levels detected in plasma, it is possible that DKK3 could serve as an indicator not only for renal disease but also for the overall condition. Although glomerular injury is not a characteristic feature of ADPKD, some patients may show relevant albuminuria. uDKK3 could hence—in principle—be derived from filtered plasma DKK3 following glomerular injury. However, currently available data point towards the fact the DKK3—due to its primary availability in complexes—is neither filtered nor found in the urine of nephrotic patients to a relevant degree [9].

A recent study was able to confirm DKK3 to identify patients with short-term risk of eGFR loss [9]. In the study, DKK3 risk progression in CKD patients of different etiologies was possible even beyond established biomarkers. To determine whether DKK3 affects routine clinical care, we decided to examine whether adding uDKK3 levels to the Mayo classification model would improve its ability to predict future eGFR. Our linear model confirms uDKK3 in improving the prediction of the eGFR slope. uDKK3 was indeed independently associated with the eGFR slope for the optimal model including htTKV and eGFR. While uDKK3 did not add to models containing TKV, our study indicates that it could be a potential alternative when combined with clinical characteristics or other serum-based biomarkers in settings with limited access to MRI volumetry. When examining linear regression models further by addition of the biomarker copeptin, which has been shown to be an independent predictor of eGFR slope [12], the R^2^ of all tested models could be enhanced. In contrast, when copeptin was added to the linear regression models, none of the tested variables remained an independent predictor. However, when examining two models containing the interaction term of uDKK3 and copeptin, it appears that the combination of uDKK3 and copeptin may be more effective than either variable alone in predicting the slope of the eGFR. This suggests that measuring uDKK3 and copeptin simultaneously may result in a more accurate prediction of the eGFR slope, thereby potentially improving patient outcomes. However, future research with larger and more diverse samples will be required to confirm and expand upon our findings.

While uDKK3 may be a useful biomarker for guiding patient selection in targeted therapies, additional evidence is necessary to determine whether incorporating uDKK3 levels into existing predictive algorithms would improve their accuracy in predicting ADPKD disease progression.

### Limitations

This work has important limitations. While we did observe significant differences between groups, it is possible that a larger sample size could reveal more nuanced or subtle differences that were not detected in our analysis. eGFR measurements were conducted at different sites which may yield a higher variance. Besides, the study results cannot be extrapolated to patients with systemic inflammation since CRP values >10 mg/L were an exclusion criterion. A potential limitation of this study is that low numbers were observed in general, specifically in the copeptin models, for different tolvaptan doses, and when examining kidney function. It should be noted that the small sample size may have reduced the statistical power of the analyses. Future larger and prospective studies combining various biomarkers implicated in ADPKD in recent years will be crucial to move towards their clinical use.

## Supplementary Material

sfad262_Supplemental_File

## Data Availability

The data underlying this article will be shared on reasonable request to the corresponding author.
