# Supplementary material for: DKK3 as a potential novel biomarker in patients with autosomal polycystic kidney disease
Source: Clin Kidney J. 2023 Oct 13;17(1):sfad262. doi: 10.1093/ckj/sfad262 (PMC10768788; doi:10.1093/ckj/sfad262)
Supplement: sfad262_Supplemental_File [file sfad262_supplemental_file.docx]

# Supplementary


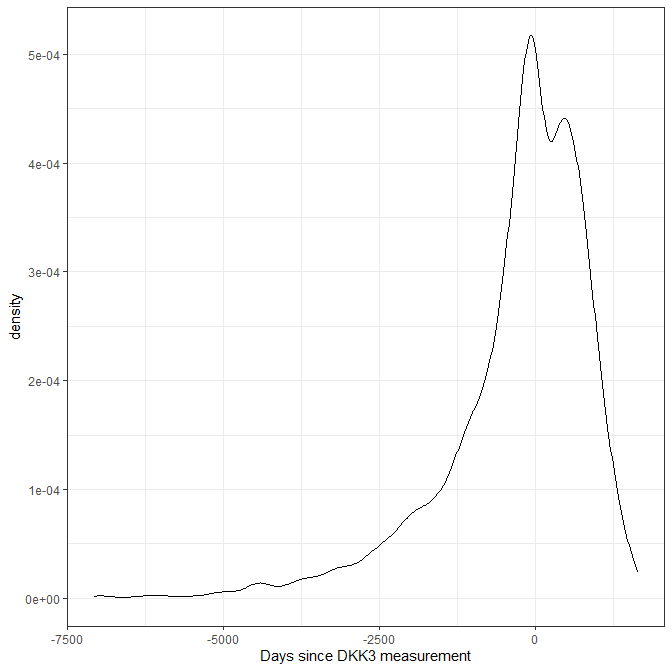


Supplemental Figure 1 Density plot displaying the relationship between historic creatinine values and days since the uDKK3 measurement.

Supplemental Figure 2 Direct comparison of uDKK3 in various age groups among ADPKD patients and healthy controls.

Supplemental Figure 3 Distribution of ADPKD patients with and without tolvaptan. A) Mayo classes and B) CKD stages. 60/30 and 90/30 indicate different dosages of tolvaptan therapy.

*Supplemental Table 1 Linear regression analysis for the age * cohort interaction term. Patients with ADPKD or healthy controls comprised cohorts.*

| Model V: log(uDKKe)~ age * cohort | | | | |
| --- | --- | --- | --- | --- |
| Adjusted R^2^ 0.1875 | | | | |
| Variable | **Estimate** | **SE** | **p-value** | **p-value summary** |
| Age | 0.04684 | 0.51791 | 1.97e-14 | *** |
| Ctrl | 0.40042 | 0.01129 | 0.7101 | ns |
| Age:Ctrl | -0.04294 | 0.02083 | 0.0407 | * |
